# Integrated Decision-Making in the Treatment of Colon-Rectal Cancer: The Case of KRAS-Mutated Tumors

**DOI:** 10.3390/life13020395

**Published:** 2023-01-31

**Authors:** Sara Cherri, Laura Melocchi, Laura Gandolfi, Giulio Rossi, Alberto Zaniboni

**Affiliations:** 1 Department of Clinical Oncology, Fondazione Poliambulanza, 25124 Brescia, Italy; 2 Department of Anatomical Pathology, Fondazione Poliambulanza, 25124 Brescia, Italy

**Keywords:** cancer, chemotherapy, colon cancer, KRAS, target therapy, multidisciplinary team

## Abstract

In recent years, precision medicine has taken an increasing place in various branches of medical oncology, including colorectal cancer. Among the potentially relevant mutations for this cancer is the KRAS mutation, initially defined as “untargetable”; today, we see the birth of new molecules that target one of the variants of the KRAS mutation, KRAS G12C, having a significant impact on the therapeutic options for other malignancies, such as metastatic lung cancer. This fundamental step forward has stimulated scientific research on other potential targets of KRAS, both indirect and direct, and combination treatments aiming to overcome the mechanisms of resistance to these drugs that decrease in efficacy in colorectal cancer. What was once a negative predictive marker of response to anti-EGFR drugs today has become a potential target for targeted treatments. In turn, the prognostic role of the mutation has become extremely interesting, making it a potentially useful element in therapeutic decision-making, not only regarding oncological treatments but also in a more complex and complete manner within a global vision of the patient, involving other figures on the multidisciplinary team, such as surgeons, radiotherapists, and interventional radiologists.

## 1. Introduction

Colorectal cancer (CRC) still represents a fundamental challenge in the oncological field today, accounting for approximately 11% of all cancer diagnoses, ranking third in terms of incidence (6.1%) and second in terms of mortality (9.2%). The relevance of this pathology is destined to increase in the coming years; in fact, it is estimated that, by 2035, the total number of deaths will increase by approximately 60% [1]. There are certainly innumerable challenges for research to address these significant data, and precision medicine is the path that has been most undertaken in recent years given the numerous goals that it has made it possible to achieve in other pathologies, such as lung and breast cancer. In colon cancers, this challenge has been found to be more complex and less satisfactory, in part due to the complex downstream signaling and numerous biological interactions implicated in colorectal cancers. Among the most important oncogenic mutations in CRC tumors is the KRAS mutation, which has always been studied but until recently was considered untargetable.

The KRAS protein is one of the rat sarcoma viral oncogene (RAS) proteins, which in turn belong to the GTPase protein family and consist of four members encoded by three genes (KRAS4a, KRAS4b, HRAS, and NRAS) that share sequence homology and differ from each other by the presence of the C-terminal hypervariable region. GTP hydrolysis following oncogenic mutations results in persistent binding and subsequent activation of downstream signaling pathways, such as Raf/MEK/ERK. Interestingly, this oncogenic mutation gives the cell continuous survival stimuli for proliferation, a phenomenon that has been identified as “KRAS addiction”.

Several codified methodologies for detecting RAS mutations are available, including direct Sanger sequencing, real-time PCR, digital PCR, pyrosequencing, mass spectrometry (Sequenom), and next-generation sequencing. The search for mutations of the RAS genes can be performed both on the primary tumor and on the metastases and recently also on plasma through so-called liquid biopsy. It is essential to have these data since oncogenic mutations in the RAS genes, as has been said, are very frequent in colorectal cancer, affecting approximately 40% of cases, of which 85% refer to the KRAS mutation, especially codons 12, 13, and 61. To date, the prognostic impact of the various KRAS mutations is not fully understood and could depend on a more complex general picture. In fact, it should be considered that, in colorectal tumors, the presence of several mutations is frequent, and the association of these mutations (for example, KRAS/p16, KRAS/p53, and others) could give the disease a distinct biological attitude that translates into a different clinical aggressiveness.

### 1.1. KRAS Mutation Determination Methods and Mutation Incidence

Mutations affecting RAS genes have long been known as early genomic events driving colorectal cancer carcinogenesis and progression in the adenoma–carcinoma sequence [2]. Considering all human tumors, the KRAS gene is the most frequently mutated (approximately 22%) among the three isoforms, followed by NRAS (8%) and HRAS (3%) [3]. Regarding mutation types, the most common genomic alterations are single nucleotide variations determining amino acid substitutions within codons 12, 13, and 61, resulting in increased affinity for GTP and constitutive activation of RAS proteins [4,5]. However, 80% of KRAS mutations are discovered within codon 12, with the remaining 20% distributed among codons 13, 59, 61, and 146, whereas mutations in NRAS are more commonly identified (60%) in codon 61 [5]. A recent Italian multi-institutional study showed KRAS and/or NRAS mutations in 47.8% of CRC cases, and among them, 90.2% harbored KRAS point mutations [6]. The most frequent KRAS mutation observed was p.G12V in exon 2 (26.4% of cases), followed by p.G12D and p.G13D detected in 19.2% and 16.5% of cases, respectively. Point mutation p.G12C was described in 8.5% of cases tested, and in another Italian cohort, it represented 17% of KRAS-mutated CRCs [7].

Since 2009, when the FDA approved first-generation EGFR inhibitors [8], several different molecular techniques for the detection of KRAS mutations have been developed, mainly in formalin/fixed paraffin embedded (FFPE) tissue. Direct Sanger sequencing has been adopted as the gold-standard method, although it is burdened by low sensitivity (20%), the need for a relatively large amount of tumor tissue DNA, and limited availability in most laboratories. In 2014, the requirement to test NRAS mutations for second-generation EGFR inhibitor eligibility [9] has indicated the need for new testing methods characterized by higher sensitivity and shorter turnaround time. At the time, pyrosequencing and the first models of allele-specific real-time PCRs (sensitivity 7–10%), requiring less tumor tissue DNA and shorter hands-on time procedures to achieve KRAS and NRAS mutations in codons 12, 13, 59, 61, 117, and 146, represented the most commonly used marketed techniques for colorectal cancer [10]. Another practice-changing study, the BEACON trial [11], made mandatory the additional testing for BRAF codon 600 mutational status as an adverse prognostic factor in EGFR-inhibitor response in monotherapy and for the eligibility of patients for BRAF inhibitors. This study led to a shift toward new molecular technologies that are able to test different target genes at the same time and from the same DNA input to contain the testing costs and the turnaround time. Then multiplexed methodologies, such as mass spectrometry and the breakthrough of next-generation sequencing (NGS), which is characterized by unprecedented sensitivity (<1%), were introduced as new routine testing methods in several tertiary centers. The need for more sensible detection tests was enhanced in 2020 by the stunning discovery of a KRAS-inhibitor molecule specifically against the p.G12C mutation [12], changing the perspective of this gene from an absolute negative predictor to a possibly positive biomarker. Along with the stunning progress of molecular technologies during the last decade, several new RT–PCR multiplexed platforms, including the CE-IVD Idylla™ KRAS and NRAS-BRAF mutation test, have been developed to detect KRAS, NRAS, and BRAF mutations in colorectal cancer patients with significantly improved specificity, sensitivity (1–5%) and rapidity of analysis. In small laboratories receiving colorectal cancer biopsies requiring all-RAS testing but lacking a real molecular facility and with no specific expertise, RT–PCR platforms, characterized by complete automation, permit reliable, and prompt results [13,14]. These easy-to-use platforms have helped to spread the RAS/RAF pathway testing possibility in a capillary fashion on the territory, enabling a larger cohort of patients to be properly treated on time. Last, plasma cell samples containing circulating tumor DNA (ctDNA) released from cancer cells represent a novel approach in detecting all-RAS oncogenic mutations in colorectal cancer, above all in the rechallenge setting [15]. Droplet digital PCR (ddPCR), NGS, and other commercially available platforms for the detection of circulating cell-free tumor DNA from liquid biopsies could show some discrepant results, but they are equally efficient for the detection of mutations in ctDNA when the mutant allelic fraction (MAF) is >5% [16,17,18] (see Table 1).

### 1.2. Predictive Impact of KRAS Mutations

Colorectal tumors are the result of the progressive accumulation of numerous genetic alterations that can involve various genes involved in the mechanisms of cell proliferation, differentiation, and survival. An important therapeutic target in the treatment of patients with mCRC is represented by drugs that target EGFR (anti-epidermal growth factor receptor), a tyrosine kinase belonging to the ErbB receptor family and implicated in cell proliferation mechanisms. Since the KRAS oncoprotein is a key player in the signaling cascade activated by EGFR, its mutation and consequent pathological activation leads to a constitutive alteration of the signal transduction mechanisms downstream of EGFR with consequent resistance to anti-EGFR treatments.

The first authors to report a reduced response to treatment in patients carrying the KRAS mutation were Lievre and colleagues in 2006, who presented a small study in which 30 patients with mCRC treated with cetuximab were screened for the KRAS mutations. This study was the first to suggest resistance to treatment in patients with KRAS mutations, thus indicating the usefulness of these data in clinical practice for the correct selection of patients who could potentially benefit from therapy with cetuximab [19]. These considerations led to an ever-growing interest in the possibility of selecting treatment in a more targeted manner, avoiding potential toxicity in patients who could be nonresponders due to the very characteristics of the disease. The confirmation of what Lievre and colleagues postulated has come from other studies that performed pooled analyses on patients treated before 2008, i.e., when the prescription of therapy with anti-EGFR drugs was not bound by the KRAS mutational status [20,21,22], and then it was confirmed by the OPUS, CRYSTAL, and PRIME studies. The OPUS and CRYSTAL studies evaluated the benefit of adding cetuximab in patients with metastatic colorectal cancer (mCRC) to the FOLFOX4 and FOLFIRI regimens, respectively, in terms of objective response rate and progression-free survival time (PFS) in the first study and in terms of overall survival (OS) and PFS in the latter. In both studies, patients with KRAS exon 2 tumor mutations did not show any benefit from adding cetuximab to the chemotherapy regimen, whereas an advantage was achieved in patients with wild-type KRAS [23,24]. These conclusions are consistent with the phase III PRIME study, which evaluated the combination of chemotherapy (FOLDFOX4) with panitumumab, the second anti-HER2 drug approved for metastatic colorectal cancer [25].

However, the predictor of response to anti-EGFR drugs also depends on other factors and is the result of the complex mechanisms involved in the cell signaling pathway and other negative predictors of response to treatment. For example, tumors that have a wild-type KRAS genotype but express a BRAF mutation or loss of EGFR/PTEN could exhibit primary resistance to targeted treatment with anti-EGFR drugs. However, currently there are no sufficiently solid data on the validity of further predictive factors of response in light of the rarity of these mutations and the difficulty in designing prospective studies that can enroll sufficient numbers of patients.

### 1.3. Prognostic Impact of KRAS Mutation

Several studies have evaluated the prognostic impact of the KRAS mutation in patients with CRC, investigating whether there was a difference in terms of prognosis compared to the type of KRAS mutation (G12C, G12V, G12D, G12A, and codon KRAS mutations 13) [26,27,28,29]. In a recent Italian study, patients harboring the KRAS G12C mutation were more likely to be men and to present with lung and liver metastases, and they were less likely to have peritoneal spread. KRAS G12C mutation was associated with shorter overall survival compared to other KRAS mutations [7]. Data on the worst relevance of one KRAS mutation compared to others are currently conflicting; however, an element that seems to have emerged from the literature is that the presence of the KRAS mutation is closely related to the patient’s prognosis in terms of disease recurrence, overall survival, and progression-free survival [30].

In a study conducted by Taieb and colleagues, in patients undergoing surgery and adjuvant chemotherapy, it seems that the KRAS mutation had a negative impact in terms of recurrence times, survival after recurrence, and overall survival only in the case of microsatellite stability; the data were not reproduced in patients with microsatellite instability [31]. Once again, this study is part of a more complex picture in which the KRAS mutation alone does not determine the eventual prognosis of patients affected by CRC, to which several factors contribute, including gender, age of the patient, staging of disease, and other molecular characteristics of tumors [32].

However, it is clear that the KRAS mutation should be considered among these factors for the global assessment of the patient’s prognosis [19]. A possible explanation can be traced back to the biological behavior of mutant KRAS diseases [33], which are generally characterized by a high incidence of vascular invasion [34], more advanced staging at diagnosis, and lymph node metastases [35].

### 1.4. KRAS Mutation in Colorectal Cancer by Tumor Sidedness

Several studies have reported that disease location based on the right or left side has a prognostic impact in terms of overall survival and progression-free survival [36,37]. It is therefore interesting to see how KRAS and sidedness data can be integrated and provide more enriched prognostic information for patients. The prognostic role of sidedness in colon tumors is related to the different genetic origins of right and left colon tumors, the different molecular profiles, and the different risk factors. In fact, the left colon originates from the hindgut, while the right colon originates from the embryonic midgut. Additionally, from an anatomical point of view, the two sides have different lymphatic and blood systems. Therefore, the tendency to treat these two pathologies as distinct pathologies has been increasingly consolidated, with worse prognostic data for patients with right colon cancer [38]. There are also several data in the literature that suggest not only a different medical approach but also a different surgical approach, with surgery that could, for example, benefit from more extensive lymphadenectomy in patients affected by right-sided mCRC [39,40]. A survey conducted in European centers reported a higher prevalence of RAS mutations in right-sided tumors than in left-sided tumors (54.6% vs. 46.4%, respectively) [41]. Despite the higher prevalence of the KRAS mutation in patients with right-sided colon cancers, it cannot be considered that the presence of the mutation alone could explain the worse prognoses of these patients. In fact, if we consider only KRAS WT patients, patients affected by right-sided tumors continue to have a worse prognosis than those affected by left-sided tumors, suggesting the presence of other implicated factors that are not known to date [42]. The guidelines provide recommendations for the treatment of colorectal tumors based on KRAS mutations regarding left-sided tumors, while less clear to date are the recommendations concerning the right colon KRAS BRAF WT, in which chemotherapy (double or triple) + the association with anti-VEGF remains the cornerstone of the treatment. Certainly, in the context of personalized oncological medicine, it will be desirable to have more molecular information from these patients to offer more treatment possibilities.

## 2. Integrated Approach

Considering what has been said until now, KRAS-mutated colorectal cancers, given the complexity of the pathology, deserve a personalized approach. This opinion implies surgical and medical considerations and more reasoned proposals for locoregional treatments. In this section, we attempt to reflect on the best medical management for mCRC patients based on the data currently available in the literature.

### 2.1. Surgery in KRAS-Mutated Colorectal Cancers

Surgery represents a therapeutic option in patients affected by colorectal cancer both in the initiated stages (I–III) defined as operable and in metastatic disease (stage IV) in selected patients, particularly in the liver, which represents the most frequent site of metastases in patients with mCRC.

With regard to operable patients, several studies have evaluated the difference in postoperative recurrence rate in patients with KRAS mutations compared to patients with wild-type KRAS. In a study conducted by Hutchins and colleagues, it was hypothesized that the mutational status of KRAS could be useful in the choice of adjuvant chemotherapy treatment in patients operated on for stage II CRC. Indeed, in this study, the presence of the KRAS mutation was associated with a higher risk of recurrence than in patients with wild-type KRAS tumors (28% (150 of 542) v 21% (219 of 1041); RR, 1.40; 95% CI, 1.12 to 1.74; *p* = 0.002) [43]. However, in a retrospective analysis of 345 patients treated for stage I–III colon cancer, of whom 40% were KRAS-mutated, KRAS mutation status was not a significant prognostic factor for disease-free survival or overall survival [44]. This result was consistent with published analyses of two randomized trials enrolling patients with stage II/III CRC treated with adjuvant chemotherapy, which concluded that the presence of KRAS mutational status did not show significant effects on survival or disease recurrence [45,46]. Interesting are data provided by a recent study that collected 150 enrolled patients and data concerning the preoperatory mutational status of KRAS based on the DNA of circulating tumor cells (ctDNA), studying its effect on the disease recurrence rate [47]. In this study, the preoperative detection of KRAS-mutated ctDNA was associated with a lower recurrence-free interval (RFI) (*p*  =  0.002) and recurrence-free survival (RFS) (*p*  =  0.025), thus suggesting that the preoperative measurement of KRAS-mutated ctDNA KRAS could be useful for deciding on postoperative treatment.

Data on the impact of the KRAS mutation have also been conflicting in patients with liver metastases. Some studies have reported a detrimental impact on overall survival in KRAS-mutated patients [30,48,49,50]; data that are not relevant have been reported in other studies published in the literature [51,52,53]. A systematic review and metanalysis conducted by Passiglia and colleagues with the aim of analyzing the outcomes of recurrence-free survival (RFS) and overall survival (OS) of patients undergoing resection of liver metastases from colon cancer predicted a significant worsening of both RFS (HR: 1.65; 95% CI: 1.23–2.21) and OS (HR: 1.86; 95% CI: 1.51–2.30) [54].

The diversity of these studies should be considered before reaching conclusions, particularly the differences in patients included in terms of other risk factors, disease staging, tumor sidedness, the presence of other risk factors, and the use of adjuvant chemotherapy treatments, which could in part explain the conflicting results.

### 2.2. Interventional Radiology Procedures in KRAS-Mutated Colorectal Cancers

The treatment of patients affected by mCRC is complex and includes a broader vision of oncology in which, next to oncologists, radiotherapists, and surgeons, there is the interventional radiologist, a fundamental figure to complete the multidisciplinary cancer team. As stated, the overall survival of patients affected by mCRC has changed over the years, and these patients can therefore afford various therapeutic opportunities, not only systemic chemotherapy but also locoregional chemotherapy treatments, within a more complex framework of care that provides maintenance and so-called stop-and-go treatments. This treatment is also made possible by locoregional treatment methods, which represent a valuable option in patients with oligo-metastases or disease progression in a single location (oligoprogression). In fact, in the first case, they allow for the treatment of a single site of disease, leading to a picture of no evidence of disease (NED) or a low burden of disease, also allowing therapeutic breaks from chemotherapy. In the second case, locoregional treatment of oligoprogression allows for continued oncological treatment, considering the response at other sites of the disease.

To date, in various centers, the use of these methods appears to be reserved as a last resort in heavily pretreated patients. This attitude is counterproductive; in fact, it is important to discuss cases in multidisciplinary teams by having colleagues evaluate the possible benefit of locoregional treatments. Several studies in the literature have pointed out the importance of an interventional radiology approach in earlier lines of treatment and in patients with still-preserved performance status. In the various interventional radiology studies in oncological patients, overall survival was not considered a good indicator since the studies suffered from serious selection bias, and they usually enrolled patients with poor performance status who were heavily pretreated and carriers of diseases with aggressive biology due to the accumulation of tumor DNA mutations [55].

In this context, it is interesting to understand whether the KRAS mutation can affect the expected response to locoregional treatment, as already postulated for patients undergoing surgery [50]. Other data in the literature have indicated that the overall survival and recurrence-free survival of patients with KRAS-mutated mCRC undergoing percutaneous ablation are significantly lower [56].

A study published in the literature by Shady and colleagues reported that the KRAS mutation could represent a significant predictor of response after radiofrequency ablation of colorectal liver metastases, thus suggesting a potential benefit of more aggressive treatments, including wider ablation zone margins for patients with KRAS-mutated mCRC, based on these results [57]. Notably, KRAS mutation was a significant predictor of OS (*p* = 0.016) (HR: 1.8; 95% CI: 1.1–2.9) and of new liver metastases (*p* = 0.037) (SHR: 2.0; CI: 1.0–3.7) and peritoneal carcinomatosis (*p* = 0.015) (sHR: 3.0; 95% CI: 1.2–7.2) in patients undergoing radiofrequency ablation of liver metastases.

These data appear to be consistent with data from a second study that evaluated whether KRAS mutational status could be a prognostic factor for survival after yttrium-90 (90Y) radioembolization for liver metastases in mCRC patients. The median OS from the first 90Y radioembolization was significantly greater in WT KRAS patients (9.5 months vs. 4.8 months; *p* = 0.041). A multivariate Cox regression analysis concluded that KRAS status is an independent prognostic factor for OS, even when correcting for the effect of chemotherapy after radioembolization [58].

### 2.3. Radiotherapy

Radiotherapy represents an important treatment in patients with colorectal cancer, especially in patients with locally advanced distal rectal tumors, defined as T3, N any with involved or threatened clear circumferential margin (by MRI); or T4, N any or locally unresectable or medically inoperable. To date, international guidelines, both NCCN and ESMO, report a pivotal role of radiotherapy in the treatment of patients with locally advanced distal rectal tumors [59,60]. Considering that approximately 40% of patients with locally advanced rectal tumors have a KRAS mutation, if this information somehow could influence the therapeutic approach in these patients, it would be of fundamental importance to consider it.

There is currently no reason to believe that patients affected by KRAS-mutated colorectal cancer have lower sensitivity to radiotherapy treatment than those in the wild-type category. In fact, it seems that the KRAS mutational status does not influence the expected response to neoadjuvant radiotherapy treatment [61,62].

A retrospective study concluded that the overall survival of patients with KRAS-mutated mCRC was significantly better in the radiotherapy + surgery group than in patients treated with surgery + chemotherapy (32 vs. 19 months, respectively). It has been reported that patients with KRAS mutations in codon 13 who were treated without radiotherapy had the lowest overall survival of all groups included in the study [63].

A multicenter phase I/II study including 18 centers in Switzerland and Hungary investigated whether the use of sotorasib concomitantly with radiotherapy could somehow increase the radiosensitivity of patients with mutant KRAS mCRC. Fifty-four patients with KRAS-mutated T3/4 and/or N1/2M0 locally advanced rectal cancers were included and treated with neoadjuvant treatment with radiotherapy (45 Gy in 25 fractions over 5 weeks) and capecitabine 825 mg/m^2^ twice daily plus sorafenib 400 mg/d. A pCR rate of 60% (95% CI, 43.3–75.1%) was reported by central independent pathologic review. Sphincter preservation was achieved in 89.5%, R0 resection in 94.7%, and downstaging in 81.6%. Grade 3 toxicities were reported, including diarrhea (15.0%), skin toxicity outside the radiotherapy field (12.5%), pain (7.5%), and cardiac ischemia (5%) [64]. These data are consistent with published results from other phase I studies [65,66].

These data are encouraging and suggest a potential different approach in neoadjuvant radiotherapy treatment in patients with KRAS-mutated, locally advanced rectal cancer.

## 3. Clinical Trials and Future Directions

**Figure 1:**
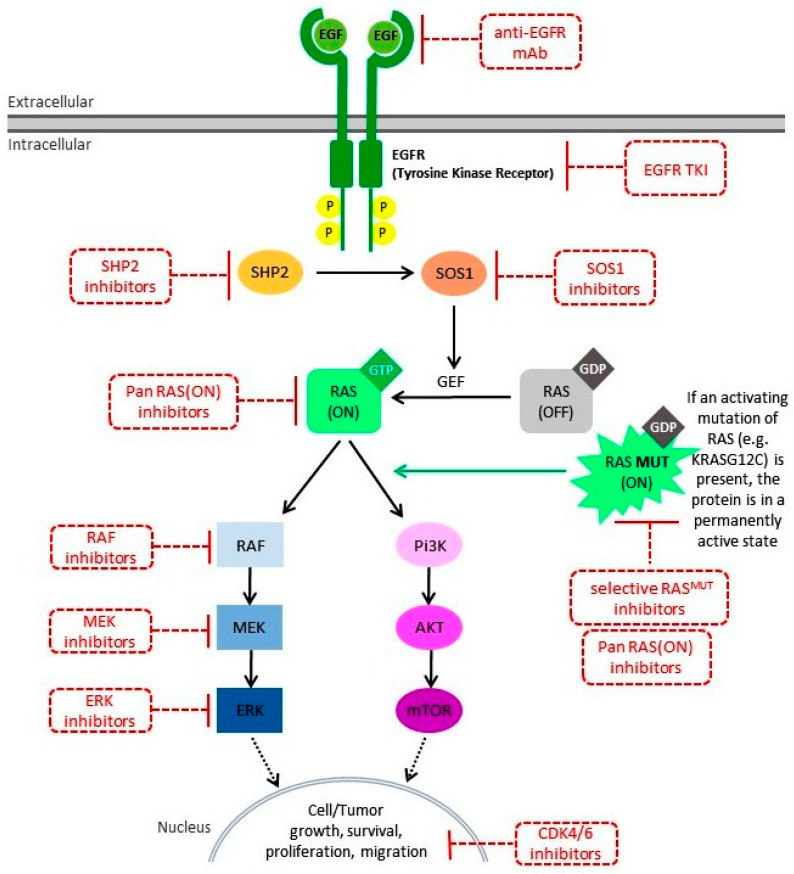
RAS signaling pathways and main potential targeting therapeutic strategies in colorectal cancer.

Considerable effort has been made in recent years to give access to precision medicine to patients with KRAS-mutated colorectal cancer. The awareness of the presence of this mutation in such a high percentage of patients and the unavailability of a targeted treatment for that mutation has always been an incentive for research with rather disappointing results, in turn leading to the consideration of the KRAS mutation as “untargetable”. To date, we can no longer consider the KRAS mutation untargetable, but there is still a long way to go. KRAS mutations have a high incidence, affecting almost half of patients with mCRC, are burdened with a worse prognosis, and are a negative predictor of response to anti-EGFR receptor antibodies. In recent years, new specific inhibitors of KRASG12C have shown promising results, especially for patients with lung cancer [67], leading to the hope of being able to replicate the same results for colorectal cancers, as well (see Figure 1).

Sotorasib is a selective inhibitor of KRAS G12C. In the CodeBreaK100 trial (NCT03600883), 62 patients with heavily pretreated KRASG12C mutant colorectal cancer were enrolled and treated with at least one dose of sotorasib. An objective response was observed in approximately 10% of enrolled patients (9.7%; 95% CI, 3.6–19.9), all with partial response [68]. Recently, similar data were published from patients with pancreatic adenocarcinoma harboring the KRAS G12C mutation [69]. Adagrasib is a covalent inhibitor of KRAS G12C that binds irreversibly and selectively to KRAS G12C. In the phase I-II KRYSTAL-1 study, adagrasib administered as monotherapy in 45 patients resulted in an overall response rate (ORR) of 22% and a disease control rate (DCR) of 87% [70]. In this same study, 22 patients receiving the combination of adagrasib plus cetuximab were enrolled, with more sustained responses with the combination therapy than with adagrasib monotherapy in terms of DOR (median duration of response) 7.6 months vs. 4.3 months, ORR (46% vs. 19%), DCR (100% vs. 86%), and PFS (6.9 months vs. 5.6 months). Recent data have been published on phase I/II study results of adagrasib with or without cetuximab in heavily pretreated patients, showing a mean response duration greater than six months in the adagrasib plus cetuximab arm [71].

The reason why the results obtained with KRASG12C inhibitor monotherapy in mCRC are less exciting than in lung cancer in terms of response to treatment and duration of response could be partly explained by the activation of different resistance mechanisms in response to the blockade of this pathway [72]. To overcome these mechanisms of resistance, several studies with combined treatments have been proposed [73]. Among these studies, very promising are combination strategies involving drug inhibitors of the KRASG12C mutation with anti-EGFR drugs. The rationale for this combination can be explained by KRAS G12C mutant CRCs retaining sensitivity to upstream RTK signaling, especially EGFR. Therefore, EGFR reactivation limits the efficacy of KRAS G12C inhibition in CRC, while its blockade with a combined KRAS G12C inhibitor and anti-EGFR approach might be effective in overcoming this adaptive resistance [72]. Data presented at the European Society for Medical Oncology meeting (ESMO 2022) from the 40-patient Phase Ib CodeBreaK 101 study dose-expansion cohort yielded a consolidation of data concerning the safety and efficacy of the combination of sotorasib and panitumumab in patients heavily pretreated with KRAS G12C-mutated metastatic CRC (Abstract 3150, cited from Annals) [74]. Most enrolled patients responded to treatment, with a tumor response of any magnitude in 88% of patients. The confirmed objective response rate (ORR) was 30% (95% confidence interval (CI): 16.6–46.5), with a disease control rate (DCR) of 93% (95% CI: 79.6–98.4). Consistently, updated data from the Phase I/II KRYSTAL-1 study confirmed previous findings. Specifically, in the adagrasib plus cetuximab combination group, the ORR was 46%, DCR was 100%, and PFS was 6.9 months (95% CI: 5.4–8.1) [71].

However, to dampen the enthusiasm for these results, it should be considered that the frequency of the KRASG12C mutation is very different between lung and colorectal cancers: 41% and 8.5%, respectively [75]. This fact has led to an effort to target other variants of KRAS mutation, KRAS G12D, the most common colon cancer-associated K-RAS mutant. Sakamoto et al. presented a K-Ras (G12D) inhibitory bicyclic peptide KS-58 that presented anticancer activity against mouse tumors derived from the colorectal cancer cell line CT26 stably expressing KRAS G12D [76]. Phase I trials are under way and could lead to an additional therapeutic possibility in patients with the KRAS mCRC mutations in the future (NCT05533463).

Obstacles to trying to directly target the KRAS mutation have led to growing research into indirect gene inhibition. In particular, the preclinical data on molecules able to target Son of Sevenless (SOS), a guanine nucleotide exchange factor that activates KRAS by catalyzing from the KRAS (off) to the KRAS (on) conformation, are very promising. In recent years, the discovery of a targetable pocket on SOS1 led to the development of SOS1 inhibitors that could have antiproliferative effects against all major KRAS mutants, thus leading to a broader concept than single mutation inhibition toward pan-KRAS inhibition [77]. Potential partners for combination with SOS1 inhibitor drugs could include MEK inhibitors, and this combination could provide a new therapeutic possibility for KRAS mutations that are not currently targetable but that represent the most widespread mutant KRAS variants, such as the G12D mutation [78]. There are several resistance mechanisms that render KRAS inhibition ineffective by acting on signaling pathways downstream of the signal, such as RAF and MEK. For example, RAF inhibition activates MEK via a feedback loop. Additionally, in this case, the combination treatment of RAF inhibitors with MEK inhibitors seems to be a potential solution to the establishment of resistance mechanisms [79]. Considering that KRAS mutations are often accompanied by mutations of oncogenes involved in the PIK3/Akt/mTOR pathway, there are numerous studies, once again of combinations, of inhibitors of this pathway, such as PI3K inhibitors and mTOR inhibitors [80]. Phase I studies are evaluating the association of sotorasib with pan-immune checkpoint inhibitors (NCT04185883). The rationale for this combination stems from the assumption that drugs binding with KRAS inhibitors should lead to sensitization of cold tumors to immunotherapy [81]. Surely an important challenge in light of the results of ongoing trials will be to understand how to select patients who can benefit from such combinations.

The next few years of clinical research will determine how much of this potential can be translated into real therapeutic possibilities for patients with mutant KRAS mCRC (see Table 2 and Table 3).

## 4. Discussion

The molecular characterization of disease has become a fundamental reference in therapeutic decision-making in different types of cancer. The study of the molecular profile, in fact, allows for the documenting of mutations involved in tumor oncogenesis and determining a response predictive factor for targeted treatments. KRAS mutation is documented in colorectal cancer in a very large number of patients, close to half of the cases, and it is considered a predictor of poor outcome [88,89]. Despite this fact, knowledge about the mutation had never been translated before into a therapeutic possibility with drugs directed against the mutation itself, which, in fact, was considered for many years to be untargetable. In clinical practice, the presence of the KRAS mutation still represents a fundamental predictor of response to treatment with anti-EGFR drugs since the presence of the KRAS mutation downstream of EGFR determines inevitable resistance to treatment. However, today, we can no longer consider this role to be the only one for the mutational state of KRAS in clinical practice. In fact, to date, this mutation is no longer considered untargetable since drug inhibitors of the KRAS G12C variant, such as sotorasib and adagrasib, are available in clinical practice.

However, this goal has not been achieved, and much remains to be accomplished for many reasons. First, the response to treatments with KRAS G12C inhibitor drugs was less promising in patients with colorectal cancer than in patients with metastatic lung cancer, likely due to the different biology of colorectal tumors, in which there are often multiple mutations and many signaling pathways involved. Furthermore, the KRAS G12C variant is less frequent in colorectal cancers, in which other variants are more represented, such as G12V, G12E, and G12A, which currently have no target treatments available. Therefore, the studies currently under way can, in fact, be divided into combination studies that associate current KRAS G12C inhibitory drugs with other drugs, with the aim of validating the resistance mechanisms and improving their outcomes; and studies with new direct inhibitors of KRAS mutation, also of different KRAS variants. Among the very relevant combination studies are association studies of KRAS G12C inhibitors with anti-EGFR monoclonal antibodies [68,70,71]. Indirect ways of inhibiting KRAS involve other leading actors of the molecular pathway, among which the most promising are SOS inhibitors (SOSi). Once again, associations of multiple inhibitors appear to be key; in the case of SOSi, the best association partners appear to be MEK inhibitors. It will be interesting to see the subsequent development of these associations, the management of toxicity, and the real applicability in clinical practice.

Molecular information about KRAS status has important implications and should be considered in a more general picture of cancer patients in different phases of the disease, and it involves different doctors on the multidisciplinary team. The presence of the KRAS mutation potentially influences many aspects of the management of cancer patients, regardless of chemotherapy and medical therapies. Several studies in the literature have investigated the impact of the mutation on various aspects of care for patients affected by CRC, both in the earliest stages and in the metastatic setting. In this article, we reviewed the integrated patient approach in terms of both medical and nonmedical cancer treatments, such as surgery, radiotherapy, and interventional radiology. Many studies have suggested greater attention and consideration in mutated KRAS patients, evaluating their responses to such treatments. Some studies have suggested a different and more invasive approach in KRAS-mutant patients, such as more extensive lymphadenectomy surgery [40] or more aggressive treatments with wider ablation-zone margins [57]. Regarding radiotherapy, several studies have evaluated, and others are currently being performed on, the benefit of combining KRASG12C inhibitory drugs with radiotherapy in the treatment of locally advanced rectal cancer [64,65,66]. These studies broaden the opportunity to select patients for more effective treatments, even in settings where the radical treatment has always been the same for everyone, regardless of the molecular status. This possibility is certainly very interesting to consider and significantly describes the change in oncological medicine in recent years.

These studies are not currently practice changing, and such data reported in the literature on this integrated approach of patients affected by mutated KRAS mCRC do not allow us to draw definitive conclusions on what the best management is for these patients to date. However, they certainly give a very positive signal of how the future of oncology is projected, and they are of fundamental importance, adding an additional step toward the conscious, complete, and as-precise-as-possible caring for the oncological patient, who is unique, as well as the disease from which he or she suffers and which we are called to cure.

## 5. Conclusions

In recent years, we have witnessed a growing interest in the predictive and prognostic significance of the KRAS mutation in colorectal cancer patients. Over the years, the possibility of using this molecular information has been enriched, ranging from the predictive value of response to anti-EGFR drugs to the possibility of accessing target therapies in the case of KRAS G12C mutations to prognostic value with potential implications in various fields. The combination of targeted treatments could be a significant therapeutic possibility in mutated-KRAS patients in the near future.

## Figures and Tables

**Table 1:** Comparison of the main key features of different molecular platforms in detecting RAS mutations.

Method	Advantages	Limitations	Tumor Sampling
Sanger sequencing	Cover all mutations with accurate results	Need for high tumor cellularity > 30% with enrichment Higher costs Low throughput Low VAF mutations may be missed.	Tissue (cytology and biopsy)
Next generation sequencing (NGS)	High sensitivity and high throughput Detection of multiple targets and multiple alteration types (mutations, amplifications, gene fusions) simultaneously with small amounts of input nucleic acids	Complex, requiring bioinformatic support Labor intensive	Tissue (cytology and biopsy) and plasma
Allele specific PCR (ARMS)	Quick and easy to use Can detect as little as 1% mutants in normal DNA background Can detect multiple specific mutations	Allele specific (only alterations targeted by the assay) False-positive results	Tissue (cytology and biopsy) and plasma
Droplet digital PCR (ddPCR)	Extremely sensitive (0.04–0.1%) Rapid TAT Less baseline DNA than sequencing methods	Allele specific (only alterations targeted by the assay)	Tissue (cytology and biopsy) and plasma
Mass array (MALDI-TOF mass spectrometry)	Rapid TAT Low cost	Allele specific (only alterations targeted by the assay)	Tissue (cytology and biopsy) and plasma
Idylla^TM^	Rapid TAT User friendly	Allele specific (only alterations targeted by the assay)	Tissue (cytology and biopsy) and plasma

Abbreviation: VAF, variant allele frequency; NGS, next-generation sequencing; PCR, polymerase chain reaction; ARMS, amplification-refractory mutation system; ddPCR, droplet digital PCR; TAT, turnaround time; MALDI-TOF, matrix-assisted laser desorption/ionization–time of flight.

**Table 2:** Concluded and ongoing clinical trials testing selective KRASG12C inhibitors in metastatic colorectal cancer (mCRC), alone or in combination (some exclusively Asian trials were excluded).

Drug	NCT Number Trial Name	Phase	KRAS MT mCRC pts	Results
Sotorasib (AMG 510) [12]	NCT03600883 CodeBreaK100	I	(a) All doses: 42 (b) Cohort that received 960 mg daily: 25	(a) ORR: 7.1% (3/42) DCR: 73.8% (31/42) mPFS: 4 months (b) ORR: 12% (3/25) DCR: 80% (20/25) mPFS: 4 months
Sotorasib (960 mg orally once per day) [68]		II	62	ORR: 9.7% (6/62, all PR)
Sotorasib + panitumumab [74]	NCT04185883 CodeBreaK101	I/II	40	ORR: 30% (12/40) DCR: 90% (37/40) DOR: 5.9 months mPFS: 5.7 months
Sotorasib + anti PD-1, MEKi, SHP2 allosteric inhibitor, pan-ErbB inhibitor, anti-PD-L1, anti-EGFR, ChT, mTORi, or CDK4/6i		I	Estimated enrollment: 1054, not only CRC	Ongoing
Sotorasib + panitumumab versus ChT (in the third line)	NCT05198934 CodeBreaK 300	III	Estimated enrollment: 153	Ongoing (Estimated primary completion date: March 3023)
Adagrasib (MRTX849) [82]	NCT03785249 KRYSTAL-1	I	2 (pts evaluable for clinical activity/4 mCRC)	ORR: 1 pts DOR: 4.2 months
Adagrasib monotherapy (600 mg BID) [70]		II	45 (pts evaluable for clinical activity)	ORR: 22% (10/45) DCR: 87% (39/45) DOR: 4.2 months mPFS: 5.6 months
Adagrasib + cetuximab [70,71]		II	28 (pts evaluable for clinical activity)	ORR: 46% (13/28) DCR: 100% (28/28) DOR: 7.6 months mPFS: 6.9 months
Adagrasib + pembrolizumab or afatinib		IB	-	Ongoing
Adagrasib + TNO155 (SHP2 inhibitor) [83]	NCT04330664 KRYSTAL-2	I/II	86	Ongoing, not recruiting
Adagrasib + BI 1701963 (SOS1 Inhibitor)	NCT04975256 KRYSTAL 14	I	7	Completed, data not available
Adagrasib + palbociclib	NCT05178888 KRYSTAL-16	I	11, not only CRC	Active, not recruiting
Adagrasib + cetuximab versus ChT (in the second line)	NCT04793958 KRYSTAL-10	III	Estimated enrollment: 420	Ongoing (Estimated primary completion date: September 2023)
Adagrasib monotherapy	NCT05162443	Expanded access	Advanced solid tumors harboring a KRAS G12C mutation	Ongoing
JNJ-74699157 [84]	NCT04006301	I	4	In overall population (10 pts) ORR, 0; DCR (SD), 4 pts Stopped due to dose-limiting toxicities and lack of efficacy
LY3499446	NCT04165031	I/II	5	Early termination due to unexpected toxicity
LY3537982 +/− abemaciclib, erlotinib, pembrolizumab, temuterkib, LY3295668, cetuximab, or TNO155	NCT04956640	I	Estimated enrollment: 360, not only CRC	Ongoing (Estimated primary completion date: November 2023)
IBI351 (GFH925) [85]	NCT05005234	I	3	1 PR, 2 PD
IBI351 + cetuximab	NCT05497336	IB	Estimated enrollment: 80	Ongoing (Estimated primary completion date: August 2023)
JAB-21822 [86]	NCT05009329	I/II	9 mCRC (33 overall population)	in the overall population at the dose of 800 mg QD: ORR: 50% (5/10) DCR: 100% (10/10)
JAB-21822 + cetuximab	NCT05194995	IB/II	Estimated enrollment: 62 (mCRC, small intestinal and appendiceal cancer)	Ongoing (Estimated primary completion date: December 2023)
JAB-21822 +/− cetuximab	NCT05002270	I/II	Estimated enrollment: 100, not only CRC	Ongoing (Estimated primary completion date: July 2023)
JAB-21822 + JAB-3312 (SHP2 inhibitors)	NCT05288205	I/II	Estimated enrollment: 124, not only CRC	Ongoing (Estimated primary completion date: March 2026)
GDC-6036 +/− atezolizumab, cetuximab, bevacizumab, erlotinib, GDC-1971, or inavolisib [87]	NCT04449874	I	Estimated enrollment: 498, not only CRC	Ongoing (Estimated primary completion date: August 2023) GDC-6036 monotherapy (43 pts): confirmed ORR 20% (8/41 pts)
JDQ443 +/− TNO155 or tislelizumab	NCT04699188 KontRASt-01	I/II	Estimated enrollment: 425, not only CRC	Ongoing (Estimated primary completion date: August 2024)
JDQ443 +/− trametinib, ribociclib, or cetuximab	NCT05358249 KontRASt-03	I/II	Estimated enrollment: 346, not only CRC	Ongoing (Estimated primary completion date: June 2025)
HBI-2438	NCT05485974	I	Estimated enrollment: 44, not only CRC	Ongoing (Estimated primary completion date: August 2025)
BPI-421286	NCT05315180	I	Estimated enrollment: 80, not only CRC	Ongoing (Estimated primary completion date: July 2023)
RMC-6291	NCT05462717	I	Estimated enrollment: 117, not only CRC	Ongoing (Estimated primary completion date: November 2024)
D-1553	NCT04585035	I/II	Estimated enrollment: 200, not only CRC	Ongoing (Estimated primary completion date: November 2022)
BI 1823911 +/− BI 1701963 (SOS1 inhibitor)	NCT04973163	I	Estimated enrollment: 72, not only CRC	Ongoing (Estimated primary completion date: July 2024)

Abbreviations: MT, mutation; pts, patients; CRC, colorectal cancer; ORR, objective response rate; DCR, disease control rate; mPFS, median progression-free survival; DOR, duration of response; CHT, chemotherapy; PR, partial response; SD, stable disease.

**Table 3:** Ongoing clinical trials testing other RAS inhibitors (alone or in combination) in solid tumors with KRAS mutations.

Drugs	Mechanism of Action	NCT Number	Phase	Status (In December 2022)
ASP3082 +/− cetuximab	Direct KRAS G12D targeting	NCT05382559	I	Recruiting
HRS-4642		NCT05533463	I	Recruiting
MRTX1133		Positive preclinical studies		
BI1701963 +/− trametinib	SOS1 inhibitors (pan-KRAS inhibitors)	NCT04111458	I	Active, not recruiting
BI1701963 + irinotecan		NCT04627142	I	Completed, data not available
BI1701963 +/− BI3011441 (MEK inhibitor)		NCT04835714	I	Completed, data not available
MRTX0902 +/− adagrasib		NCT05578092	I/II	Recruiting
RMC-4630	SHP2 inhibitors (pan-KRAS inhibitors)	NCT03634982	I	Active, not recruiting
RMC-4630 + sotorasib		NCT05054725	II	Recruiting
RMC-4630 + LY3214996 (ERK1/2 inhibitor)		NCT04916236 SHERPA	I	Recruiting
RMC-4630 +/− cobimetinib or osimertinib		NCT03989115	I/II	Completed, data not available
TNO155		NCT03114319	I	Recruiting
JAB-3068		NCT03518554	I	Recruiting
JAB-3068 +/− PD1 inhibitors		NCT04721223	I/II	Recruiting
JAB-3312		NCT04045496	I	Recruiting
JAB-3312 + binimetinib, pembrolizumab, sotorasib, or osimertinib		NCT04720976	I/II	Recruiting
RLY-1971 (other names: GDC-1971, RO7517834)		NCT04252339	I	Active, not recruiting
GDC-1971+ atezolizumab		NCT05487235	I	Recruiting
BBP-398		NCT05621525 NCT04528836	I	Recruiting
BBP-398 + sotorasib		NCT05480865 Argonaut	I	Recruiting
BBP-398 + nivolumab		NCT05375084	I	Recruiting
ERAS-601 +/− cetuximab or pembrolizumab		NCT04670679 FLAGSHP-1	I	Recruiting
SH3809		NCT04843033	I	Recruiting
PF-07284892 (other name: ARRY-558) +/− lorlatinib, encorafenib, and cetuximab or binimetinib		NCT04800822	I	Recruiting
SAR442720 + pembrolizumab or adagrasib		NCT04418661	I/II	Recruiting
RMC-6236	RASMUTLI(ON) inhibitor (pan RAS(ON) inhibitor), selective for the active RAS(ON) form of both wild-type and mutant variants of the canonical RAS isoforms (HRAS, NRAS, and KRAS)	NCT05379985	I	Recruiting
RSC-1255	Pan-mutant and wild-type RAS inhibitor	NCT04678648	I	Recruiting

## Data Availability

Data sharing not applicable.
